# Rhinoscleroma Masquerading as a Nasal Mass: A Diagnostic Challenge in a Young Adult

**DOI:** 10.7759/cureus.108774

**Published:** 2026-05-13

**Authors:** Rutuja Pandharmise, Vivek Harkare, Sonali P Khadakkar, Fahad I Idrees, Sanaya D Dholkawala

**Affiliations:** 1 Otolaryngology - Head and Neck Surgery, N. K. P. Salve Institute of Medical Sciences and Research, Nagpur, IND; 2 Medical Education, N. K. P. Salve Institute of Medical Sciences and Research, Nagpur, IND

**Keywords:** granulomatous disease, klebsiella rhinoscleromatis, nasal airway obstruction, nasal cavity mass, rhinoscleroma

## Abstract

Rhinoscleroma is a rare, chronic granulomatous disease of the upper respiratory tract caused by Klebsiella rhinoscleromatis. Although uncommon, it continues to persist in developing regions and often presents a diagnostic challenge due to its indolent course, nonspecific clinical features, and resemblance to other granulomatous or neoplastic conditions.

We report the case of a 23-year-old male who presented with progressively worsening bilateral nasal obstruction, intermittent epistaxis, and persistent mucopurulent nasal discharge over a duration of six months. Endoscopic evaluation revealed multiple firm nodular lesions involving the nasal vestibule, septum, and extending to the hard palate, suggesting locally aggressive yet non-destructive pathology.

Initial histopathological examination was inconclusive, demonstrating only nonspecific inflammatory changes. However, a repeat biopsy combined with immunohistochemical analysis confirmed the diagnosis. Radiological imaging showed a heterogeneously enhancing soft tissue lesion with smooth bony remodeling leading to near-total nasal obstruction, without evidence of bone destruction.

The patient was managed with a combination of systemic corticosteroids and prolonged antimicrobial therapy, resulting in significant clinical improvement and regression of lesions. This case underscores the importance of maintaining a high index of suspicion in endemic areas, the diagnostic value of repeat biopsy, and the necessity of prolonged therapy to prevent recurrence and complications.

## Introduction

Rhinoscleroma is a slowly progressive granulomatous infection primarily affecting the nasal cavity, with potential extension to the nasopharynx, larynx, trachea, and bronchi [1]. It represents a chronic inflammatory condition characterized by granuloma formation followed by fibrosis, which may ultimately result in structural deformity and airway compromise [2].

The causative organism, Klebsiella rhinoscleromatis, is a Gram-negative, encapsulated bacillus belonging to the Enterobacteriaceae family; the disease is endemic in regions with low socioeconomic conditions, particularly in parts of Asia, Africa, Eastern Europe, and Central America [3]. Predisposing factors include overcrowding, poor hygiene, malnutrition, and limited access to healthcare facilities. Transmission is thought to occur via inhalation of infected droplets, with young adults being the most commonly affected population [3].

Clinically, rhinoscleroma is notorious for its insidious onset and prolonged course. Patients often present with nonspecific symptoms such as nasal obstruction, crusting, discharge, and occasional bleeding [4]. As the disease progresses, it may mimic other granulomatous diseases like tuberculosis, fungal infections, and granulomatosis with polyangiitis, as well as malignancies of the nasal cavity [5]. This clinical overlap frequently leads to delayed diagnosis and mismanagement.

Given these challenges, histopathological examination remains the cornerstone of diagnosis, although repeated biopsies may be necessary in early or atypical cases [5]. This report aims to highlight a diagnostically challenging case and emphasize the importance of clinical suspicion and appropriate investigative strategies.

## Case presentation

A 23-year-old male presented to the outpatient department with complaints of progressive bilateral nasal obstruction, intermittent episodes of epistaxis, and persistent mucopurulent nasal discharge for a period of six months. The symptoms were gradual in onset and had progressively worsened, significantly affecting his quality of life. There was no associated history of fever, weight loss, trauma, prior nasal surgery, or systemic illness.

**Figure 1:**
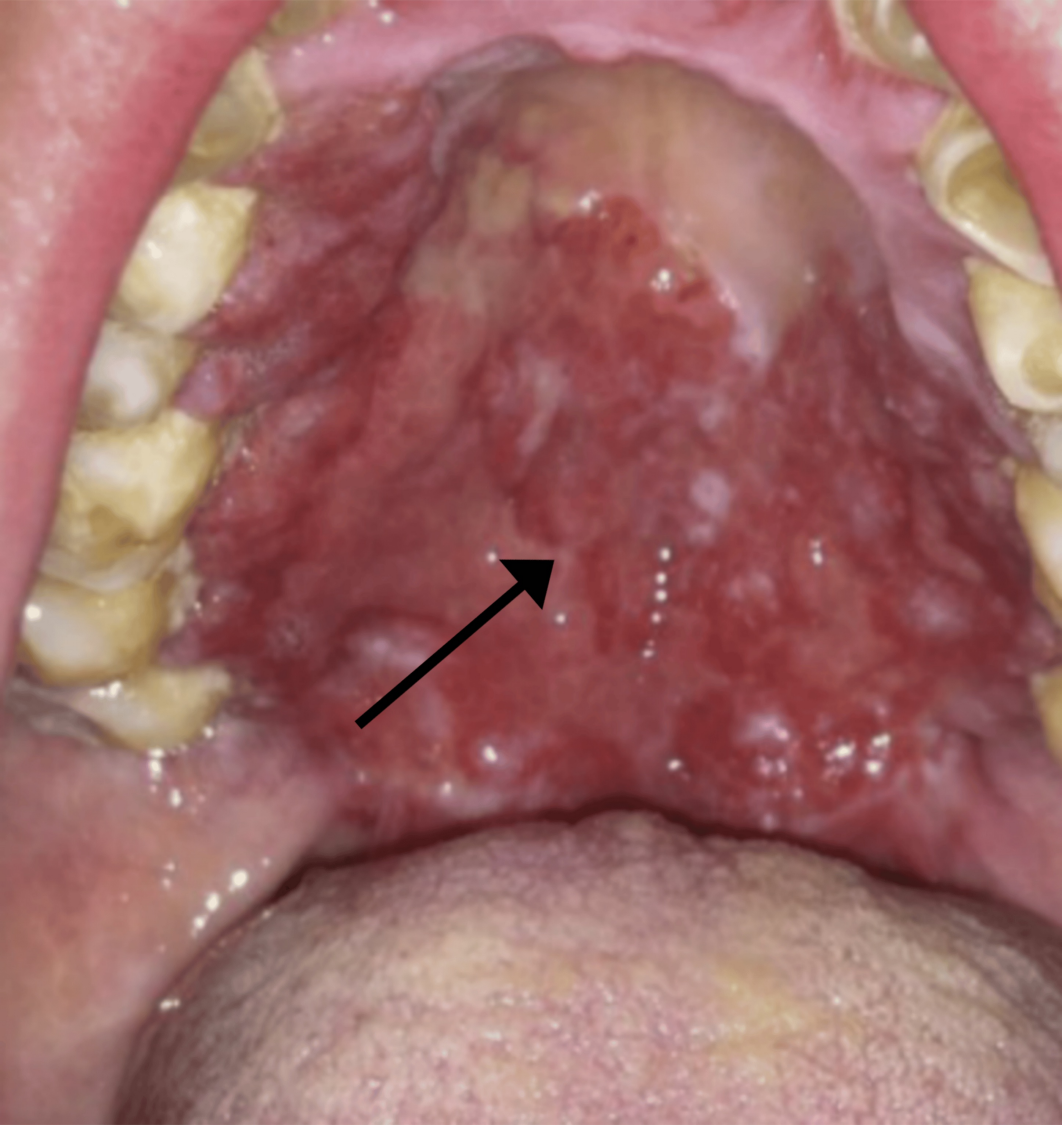
Intraoral view showing erythematous nodular mucosal lesions involving the hard palate, with areas of surface irregularity and focal ulceration (arrow).

**Figure 2:**
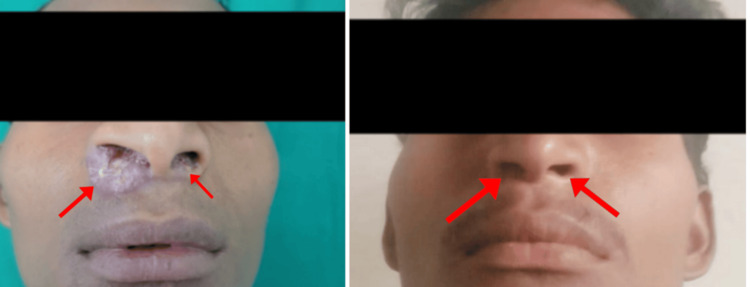
Pre-treatment clinical image demonstrating ulcerative, crusted lesions involving the nasal ala and nasal vestibule, with associated mucosal irregularity and inflammatory changes (A, arrows), and post-treatment image showing resolution of the lesions involving the nasal ala and nasal vestibule (B, arrows).

Anterior rhinoscopic examination revealed irregular nodular masses occupying both nasal cavities. Diagnostic nasal endoscopy demonstrated multiple firm, non-tender nodular lesions involving the nasal vestibules and septum, extending along the floor of the nasal cavity. Additionally, similar lesions were observed over the hard palate, suggesting contiguous spread of the disease process (Figure 1). Pre-treatment clinical examination showed ulcerative, crusted lesions involving the nasal ala and nasal vestibule, with associated mucosal irregularity and inflammatory changes (Figure 2A). 

**Figure 3:**
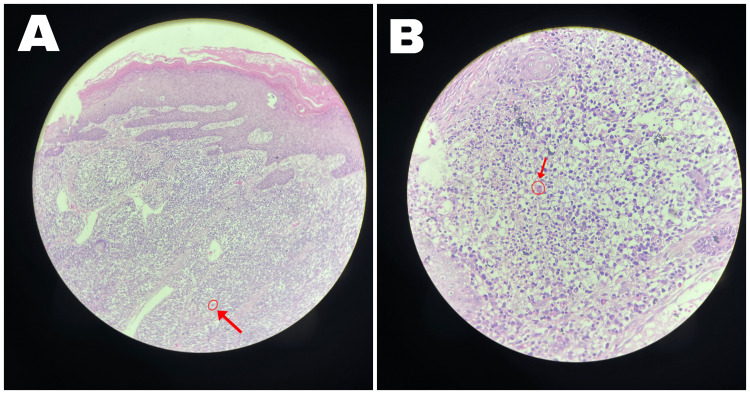
Histopathological features of rhinoscleroma on hematoxylin and eosin (H&E) staining. (A) Low-power photomicrograph (×40) demonstrating stratified squamous epithelium with a dense subepithelial chronic inflammatory infiltrate composed predominantly of lymphocytes and plasma cells. (B) High-power photomicrograph (×100) showing numerous plasma cells with prominent eosinophilic intracytoplasmic inclusions (Russell bodies) (arrow), consistent with chronic granulomatous inflammation in rhinoscleroma.

An initial biopsy performed elsewhere revealed nonspecific chronic inflammatory changes and was inconclusive. Repeat biopsy revealed sheets of foamy macrophages (Mikulicz cells) containing suspected intracellular bacillary structures, along with numerous plasma cells and Russell bodies, findings consistent with rhinoscleroma (Figure 3). Immunohistochemical analysis demonstrated positivity for CD68, highlighting histiocytes (Mikulicz cells), supporting the diagnosis. Immunohistochemistry (IHC) was supportive but not the primary basis for the diagnosis.

**Figure 4:**
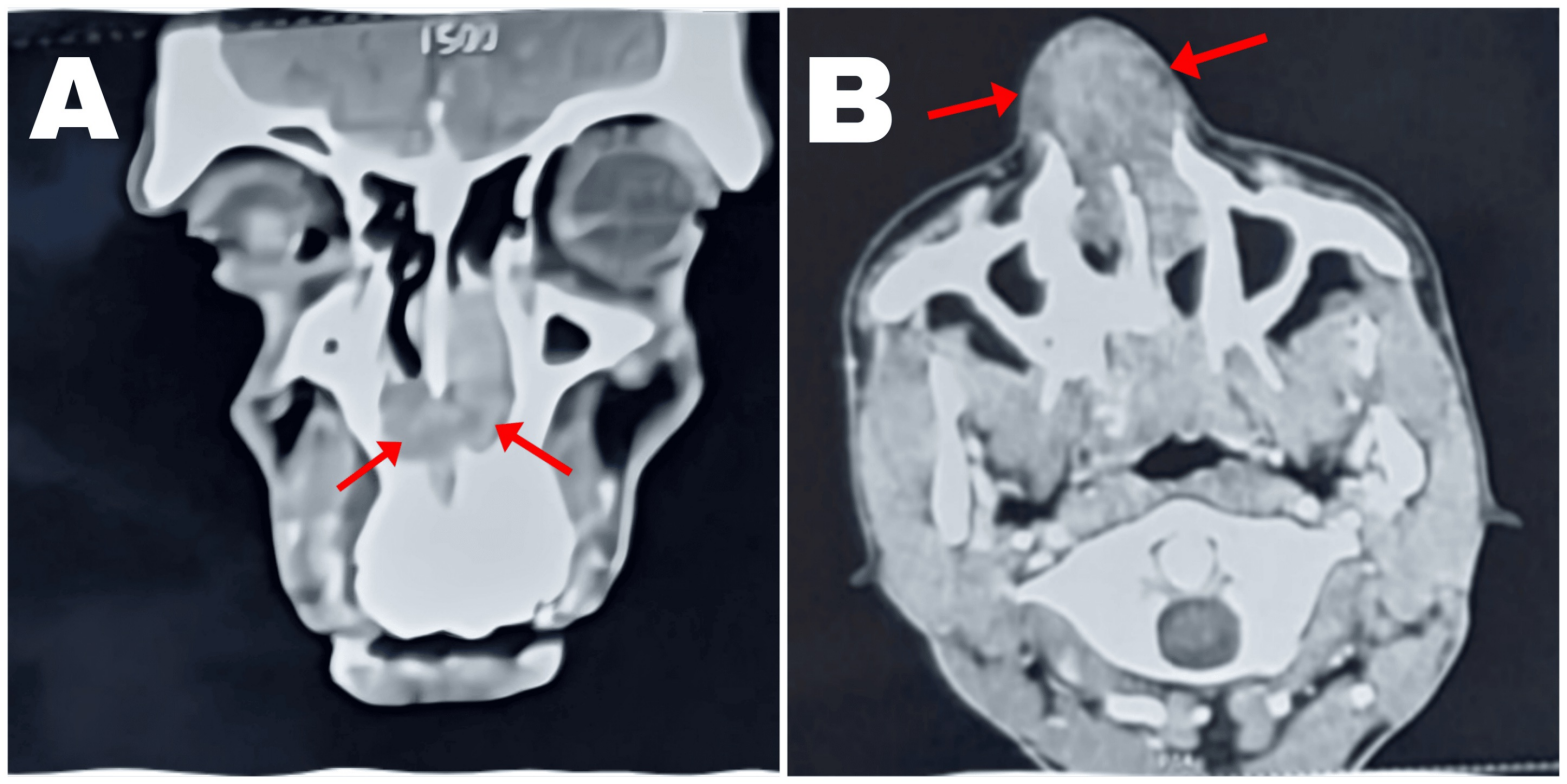
Contrast-enhanced computed tomography (CT) scan of the paranasal sinuses. (A) Coronal section demonstrating near-complete obliteration of the bilateral nasal cavities (arrows). (B) Axial section showing a soft-tissue density lesion causing marked nasal cavity obstruction with extension into the anterior nasal region (arrows).

Contrast-enhanced computed tomography (CT) scan of the paranasal sinuses revealed an irregular, heterogeneously enhancing soft tissue mass causing near-complete obstruction of the nasal cavities. Notably, there was smooth bony remodeling without evidence of bone destruction, favoring a chronic inflammatory etiology over malignancy (Figure 4; Table 1).

**Table 1:** Laboratory investigations and differential diagnostic workup. Laboratory investigations were largely unremarkable, with mild elevation of inflammatory markers. Workup for infectious and autoimmune differentials was negative.

Investigations	Parameter	Patient value/status	Reference range	Clinical significance
Complete blood count	Hemoglobin (Hb)	Within normal limit (13.8 g/dL)	13-17 g/dL	Assesses anemia
Complete blood count	Total leukocyte count (TLC)	Within normal limit (9,500 cells/mm^3^)	4,000-11,000 cells/mm^3^	Indicates infection/inflammation
Complete blood count	Differential leukocyte count (DLC)	Within normal limit N: 55%, L: 28%	Neutrophils (N): 40%-70%, lymphocytes (L): 20%-40%	Identifies infection pattern
Inflammatory marker	Erythrocyte sedimentation rate (ESR)	Mildly elevated (17 mm/hour)	0-15 mm/hour	Chronic inflammation
Inflammatory marker	C-reactive protein (CRP)	Mildly elevated (7.2 mg/L)	<6 mg/L	Active inflammation
Tuberculosis workup	Mantoux test	Negative	< 5 mm induration	Rules out Tuberculosis
Tuberculosis workup	Chest X-ray	Normal	-	Excludes pulmonary tuberculosis
Sarcoidosis marker	Serum angiotensin converting enzyme (ACE)	Within normal limit (34 U/L)	8-52 U/L	Excludes sarcoidosis
Autoimmune marker	Cytoplasmic anti-neutrophil cytoplasmic antibodies (c-ANCA)	Negative	Negative	Rules out granulomatosis with polyangiitis
Fungal workup	Potassium hydroxide (KOH) mount	Negative	No fungal elements	Excludes fungal infection
Fungal workup	Fungal culture	No growth	No growth	Confirms absence of fungus

Systemic corticosteroids were used as an adjunct to reduce the inflammatory bulk and relieve obstruction before antimicrobial therapy. Ciprofloxacin was initiated at a dose of 500 mg twice daily due to its intracellular penetration and activity against Klebsiella rhinoscleromatis. Subsequently, rifampicin 600 mg once daily and streptomycin 1 g intramuscularly once daily were administered for six weeks based on availability and institutional protocol. Although longer durations are recommended in the literature, a six-week course resulted in significant clinical improvement in this case. At the three-month follow-up, the patient remained symptom-free with no evidence of recurrence.

## Discussion

Rhinoscleroma remains a rare yet clinically important entity, particularly in endemic regions where it continues to pose diagnostic and therapeutic challenges; the disease typically progresses through three overlapping stages: the catarrhal (atrophic), granulomatous, and sclerotic phases, although overlap between phases may occur [3,5]. Unlike malignancies, rhinoscleroma typically demonstrates smooth bony remodeling rather than destruction, lacks cytological atypia on histology, and shows characteristic Mikulicz cells, aiding differentiation.

The initial catarrhal stage is characterized by nonspecific symptoms such as nasal discharge, crusting, and mild obstruction, often leading to misdiagnosis. The granulomatous stage, as seen in this case, involves the formation of nodular masses that can cause significant obstruction and local tissue involvement. In the late sclerotic phase, fibrosis and scarring predominate, potentially resulting in airway stenosis and permanent deformities [6].

Histopathology remains the gold standard for diagnosis; the presence of Mikulicz cells (foamy macrophages containing bacteria) and Russell bodies (plasma cell inclusions) is characteristic. Special stains such as PAS or Warthin-Starry were not performed in this case, representing a limitation of the diagnostic workup. However, early lesions may lack these classical features, making diagnosis difficult and necessitating repeat biopsy, as demonstrated in this case [7]. Although tissue culture was not performed in this case, the diagnosis was supported by characteristic histopathological, immunohistochemical, clinical, and radiological findings.

Radiological imaging, particularly CT scans, plays an important supportive role by delineating the extent of disease and differentiating it from malignancies, which include typical findings such as soft tissue masses with homogeneous or heterogeneous enhancement and smooth bone remodeling, rather than aggressive bone destruction [6].

Management primarily involves prolonged antibiotic therapy. Drugs such as rifampicin, fluoroquinolones, and aminoglycosides are effective due to their ability to penetrate intracellularly and target the causative organism [8]. Combination therapy is often preferred to reduce recurrence rates. Corticosteroids may be used as adjunctive therapy to control inflammation and reduce lesion size. However, corticosteroids are not routinely recommended as first-line therapy and should be used judiciously. Surgical intervention is reserved for cases with significant obstruction, deformity, or complications [8].

Prolonged antibiotic therapy remains the cornerstone of treatment, often extending over several months to reduce recurrence. Commonly used agents include rifampicin, fluoroquinolones, and tetracyclines due to their intracellular activity. In our case, a shorter duration was used with favorable short-term outcomes; however, longer follow-up is required, given the known risk of relapse. Given the high relapse rate of rhinoscleroma, close follow-up is essential. In our case, the patient remained disease-free at 3-month follow-up; however, longer surveillance is warranted. Although some authors recommend continuation of therapy until repeated cultures or biopsies become negative, follow-up microbiological or histopathological reassessment was not performed in our case. Treatment cessation was based on marked clinical and endoscopic improvement, with no evidence of recurrence at short-term follow-up. Potential complications include airway stenosis, septal perforation, nasal deformity, and, in rare cases, malignant transformation [9]. This case highlights the importance of considering rhinoscleroma in the differential diagnosis of chronic nasal masses and reinforces the need for repeated histopathological evaluation when initial findings are inconclusive.

## Conclusions

Rhinoscleroma, although rare, should be considered in patients presenting with chronic nasal obstruction and mass lesions, especially in endemic regions or in the presence of risk factors such as poor socioeconomic conditions.

This case emphasizes the critical role of clinical suspicion and the importance of repeat biopsy in establishing a definitive diagnosis when initial investigations are inconclusive. Early and accurate diagnosis allows timely initiation of appropriate therapy, which primarily consists of prolonged antimicrobial treatment.

A multidisciplinary approach involving clinicians, radiologists, and pathologists is essential for optimal management. Prompt intervention not only alleviates symptoms but also prevents disease progression, recurrence, and long-term complications, thereby significantly improving patient outcomes.

## References

[1] Saout Arrih B, Marouf R, Bijou W, Abada R, Mahtar M (2025). Pediatric rhinoscleroma of the nasal cavity: a case report and review of literature. Int J Surg Case Rep.

[2] Domanski MC, Rivero A, Kardon DE (2013). Rhinoscleroma presenting as a nasal-palatal mass with airway obstruction. F1000Res.

[3] Corelli B, Almeida AS, Sonego F (2018). Rhinoscleroma pathogenesis: the type K3 capsule of Klebsiella rhinoscleromatis is a virulence factor not involved in Mikulicz cells formation. PLoS Negl Trop Dis.

[4] Kabila B, Zhim M, Naggar A (2023). Rhinoscleroma in a 9-year old boy: rare case report. Glob Pediatr Health.

[5] Malkud S, Mahajan P (2018). Rhinoscleroma: an unusual presentation. Indian Dermatol Online J.

[6] Ibrahim D, Fayed A (2018). Report of a case of giant rhinoscleroma: CT and MRI. BJR Case Rep.

[7] Efared B, Hammas N, Gabrielle AE, Ben Mansour N, El Fatemi H, Chbani L (2018). Rhinoscleroma: a chronic infectious disease of poor areas with characteristic histological features - report of a series of six cases. Trop Doct.

[8] Bazzout A, Lachkar A, Benfadil D, Tsen AA, El Ayoubi F, Ghailan R (2021). Rebellious headache revealing an extensive rhinoscleroma: a case report and review of the literature. Ann Med Surg (Lond).

[9] de Pontual L, Ovetchkine P, Rodriguez D (2008). Rhinoscleroma: a French national retrospective study of epidemiological and clinical features. Clin Infect Dis.

